# Understanding mHealth Engagement Among Patients With 30-Day Hospital Revisits: Secondary Analysis of a Randomized Clinical Trial

**DOI:** 10.2196/89067

**Published:** 2026-05-29

**Authors:** Susan Landon, Angira Mondal, Aiden Ahn, Klea Profka, Anna U Morgan, Eric Bressman

**Affiliations:** 1 Department of Medicine Perelman School of Medicine University of Pennsylvania Philadelphia, PA United States; 2 Leonard Davis Institute of Health Economics University of Pennsylvania Philadelphia, PA United States; 3 Center for Health Incentives and Behavioral Economics University of Pennsylvania Philadelphia, PA United States; 4 Department of Medical Ethics and Health Policy University of Pennsylvania Philadelphia, PA United States; 5 Philadelphia VA Medical Center Philadelphia, PA United States

**Keywords:** mobile health, mHealth, readmissions, transitional care management, quality improvement

## Abstract

**Background:**

Reducing 30-day hospital readmissions has been a long-standing goal across health systems in the United States. While nurse-led phone outreach has been widely adopted to support transitional care, its reach is constrained by staffing and time limitations. Mobile health (mHealth) interventions, such as automated SMS text messaging and patient portals, offer scalable alternatives but have shown mixed effectiveness in reducing readmissions. Understanding how patients engage with mHealth after discharge may help optimize these tools for postdischarge care.

**Objective:**

This study aimed to characterize patients in an mHealth transitional care program who experienced hospital revisits within 30 days of discharge, comparing demographic and clinical characteristics, intervisit interactions, and revisit features between those who engaged with mHealth and those who did not.

**Methods:**

We conducted a secondary analysis of patients in the intervention arm of the Mobile Outreach to Reduce Emergencies-Primary Care randomized clinical trial. Participants received automated SMS text messages for 30 days after discharge alongside usual transitional care. We identified patients with a 30-day hospital revisit and conducted manual chart reviews to assess mHealth engagement and other forms of health care contact. We compared patient characteristics, intervisit interactions, and revisit features (time to revisit, relatedness to index hospitalization, and predictability of revisit) between mHealth users and nonusers.

**Results:**

Among 496 patients with a 30-day revisit, 185 (37%) engaged with mHealth before their return. mHealth users were younger (n=47, 26% aged <50 years vs n=41, 14% among nonusers; *P*=.004) and more likely to have commercial insurance (n=43, 23% mHealth users vs n=35, 11% mHealth nonusers; *P*=.005). Revisits were more likely to be rated highly or somewhat predictable among mHealth users compared to nonusers (n=105, 56% vs n=152, 49%; *P*=.04), while relatedness to the index hospitalization was similar (n=98, 53% vs n=173, 55%; *P*=.09). mHealth users had a longer mean time to revisit than nonusers (15.2, SD 8.1 vs 11.3, SD 8.4 days; *P*<.001) and were more likely to contact their practices via telephone (n=100, 54% vs n=136, 44%; *P*=.03) or attend a clinic visit (n=112, 61% vs n=131, 42%; *P*<.001).

**Conclusions:**

Among patients enrolled in an mHealth postdischarge program who experienced hospital revisits, fewer than half engaged with mHealth prior to their return. Revisits among mHealth users occurred later and were more predictable, suggesting that engagement may enhance situational awareness but not necessarily prevent revisits. Future work should focus on strategies to increase engagement across groups and integrate mHealth with existing transitional care infrastructure.

## Introduction

In 2012, the Centers for Medicare and Medicaid Services introduced the Hospital Readmissions Reduction Program, creating financial penalties for hospitals with elevated 30-day readmission rates and formalizing readmissions as a core quality metric [1,2]. Despite this policy, between 2016 and 2020, hospital readmission rates remained relatively steady around 14% [3]. Nonetheless, in an effort to reduce readmissions, health systems have focused on improving transitions of care, recognizing that these points in time are particularly vulnerable to error and miscommunication [4]. To date, health systems have largely focused on nurse-led phone outreach to strengthen transitional care management, but the reach of such programs is limited by staffing and time constraints [1,5,6].

Opportunities for remote interactions, however, extend beyond telephone calls. Mobile health (mHealth), defined by the World Health Organization as “medical and public health practice supported by mobile devices, such as mobile phones, patient monitoring devices, personal digital assistants, and other wireless devices,” offers a scalable alternative [7]. With expanding technology capabilities, mHealth merits particular attention for improvement initiatives. During transitions of care, mHealth interventions may take the form of, for example, a mobile app, web-based application, SMS text messaging, or remote monitoring [8,9]. Early studies of postdischarge mHealth interventions suggest possible modest readmission reductions [10-12]. They have also shown potential for improving adherence to postdischarge medication regimens and follow-up instructions, as well as increased patient-reported outcomes during the transitional care period [13,14]. In addition to aiding in adherence, it is possible that mHealth interventions may aid in early identification of complications or clinical deteriorations in transitional care periods. Particularly in cases where an index hospitalization and readmission are directly related, enhanced communication between patients and providers may help to increase predictability of a possible readmission with the intention of identifying potential avenues for intervention to avoid hospital revisits where feasible.

To overcome barriers of call-based strategies, the Mobile Outreach to Reduce Emergencies-Primary Care (MORE-PC) trial, one of the largest postdischarge mHealth trials to date, tested implementation of an SMS text messaging–based platform to reduce 30-day readmissions, showing no significant reduction in readmissions [15]. In this secondary analysis, we study patients in the intervention arm with 30-day hospital revisits. Comparing patients who used mHealth and patients who did not, our objectives were to analyze patient characteristics, interaction patterns leading up to revisits, and revisit characteristics to inform future development of mHealth transitional care interventions.

## Methods

### Study Design

We conducted a subgroup analysis using data from the MORE-PC randomized clinical trial and manual chart review. Study reporting followed the STROBE (Strengthening the Reporting of Observational Studies in Epidemiology) guidelines (Multimedia Appendix 1) [16].

### Setting

The MORE-PC trial took place between March 2022 and January 2023 within an academic health system in Philadelphia, Pennsylvania. Participants in the intervention arm received automated SMS text messages for 30 days alongside standard transitional care management. Participants in the control arm received standard transitional care management. Full details on the study design can be found in the original manuscript [15].

### Participants

Patients included in the MORE-PC trial were adults who received care within a University of Pennsylvania Health System’s primary care practice and had been discharged home from any regional acute care facility. Patients in the intervention arm who had a hospital revisit (return emergency department [ED] visit or readmission) within 30 days of index discharge were included. We included all patients who met these criteria in our analyses.

### Ethical Considerations

This study was considered a part of the larger MORE-PC study, which was reviewed and approved by the University of Pennsylvania Institutional Review Board (protocol number 24-0431). The trial was conducted under a waiver of informed consent based on meeting criteria that both arms receive usual care, SMS text messages involved no more than minimal risk to patients, patients were free to opt out at any time after enrollment, and requiring informed consent would limit external validity of the study [15]. All data were stored on the Penn network to protect privacy. In addition, following completion of chart reviews, patient identifiers were removed for subsequent analyses. Trial participants received no compensation.

### Variables, Data Sources, and Measurement

The primary independent variable of interest was mHealth engagement, defined as using the MORE-PC SMS text messaging platform or patient portal, identified through MORE-PC data and patient charts. We used data collected on patients’ age, sex, race, ethnicity, insurer, and comorbidities from MORE-PC data files. Data on intervisit health care interactions—mHealth use, phone calls, and clinic visits—were collected from MORE-PC data files and manual chart review. We also assessed 3 primary outcome variables: time to revisit, relatedness of index hospitalization, and revisit and predictability of revisit. Time to revisit was calculated as days from discharge to revisit and was a classified as a continuous variable. Relatedness and predictability were classified as ordinal variables. Relatedness was defined as any connection between index and revisit diagnoses, as determined through chart review. For example, 2 visits for decompensated heart failure would be considered directly related. Similarly, an index visit for deep vein thrombosis and revisit for gastrointestinal bleed after starting anticoagulation would be considered directly related. An admission for pancreatitis and an admission for alcohol withdrawal would be considered indirectly related as alcohol use disorder underlies both presentations. Predictability describes the extent to which a revisit could have been anticipated based on information available to the outpatient team through review of medical records considering issues remaining at discharge and concerns brought up between visits. For example, if a patient was discharged after experiencing a bleeding gastrointestinal ulcer, reported recurrence of blood in the stool at a follow-up primary care visit, then represented to the ED, this would be considered predictable. By contrast, if a patient with an index hospitalization for pyelonephritis who had no noted complications posthospitalization, then represented to the ED for an acute stroke, this would be considered unpredictable.

### Chart Review

We developed a template for systematically reviewing and extracting data from electronic health records of the study population (Multimedia Appendix 2) in an effort to minimize bias in this process. We tested the template on 15 records to identify areas of ambiguity. Subsequently, 3 researchers (SL, AA, and KP) reviewed the same 5 records to ensure agreement across data elements. Remaining records were divided among these researchers for data extraction.

Classification of relatedness and predictability were determined through review of index visit, intervisit, and revisit documentation.

### Statistical Methods

We examined patient-level and encounter-level characteristics, comparing those engaged in mHealth and those not engaged. We assessed differences in baseline characteristics and outcome variables across groups using Wilcoxon tests for continuous variables and χ²or Fisher exact tests for categorical variables. For comparing intervisit interactions across groups, we performed 2-sample proportion testing. Patients with missing data for a given variable were excluded for that analysis. Analyses were conducted using SAS Studio (version 3.81; SAS Institute) and R software (version 4.3.2; R Foundation for Statistical Computing).

## Results

### Overview

A total of 496 patients in the intervention arm of the MORE-PC trial had a hospital revisit within 30 days of discharge. Of these, 296 (60%) were female, 217 (44%) were aged ≥71 years, 283 (57%) were Black, and 315 (64%) were insured by Medicare (Table 1). A total of 185 (37%) patients engaged with mHealth prior to their revisit.

**Table 1:** Characteristics of patients with 30-day hospital revisits and revisit-related outcomes, overall and stratified by mobile health (mHealth) engagement (N=496).

			Overall	Engaged in mHealth (n=185)	Not engaged in mHealth (n=311)	*P* value	
**Demographic characteristics**							
	Female, n (%)		296 (60)	106 (57)	190 (61)	.40	
	**Age group (years), n (%)**						.004
		<30	15 (3)	7 (4)	8 (3)		
		31-50	73 (15)	40 (22)	33 (11)		
		51-70	192 (39)	70 (38)	122 (39)		
		≥71	217 (44)	69 (37)	148 (48)		
	**Race, n (%)**						.54
		Black	283 (57)	98 (53)	185 (60)		
		White	187 (38)	78 (42)	109 (35)		
		Asian or Pacific Islander	3 (1)	1 (1)	2 (1)		
		Other	23 (5)	8 (4)	15 (5)		
	**Ethnicity, n (%)**						.59
		Hispanic	16 (3)	7 (4)	9 (3)		
		Non-Hispanic	480 (97)	178 (96)	302 (97)		
	**Insurance, n (%)**						.005
		Medicare	315 (64)	109 (59)	206 (66)		
		Medicaid	95 (19)	31 (17)	64 (21)		
		Private	78 (16)	43 (23)	35 (11)		
		Other	8 (2)	2 (1)	6 (2)		
**Clinical characteristics**							
	Comorbidity score^a^, µ (SE)		5.22 (3.0)	5.13 (3.1)	5.27 (3.0)	.50	
	Index length of stay, µ (SE)		5.51 (12.6)	5.04 (5.2)	5.79 (15.4)	.75	
	**Acute care visits within 12 months before index, µ (SE)**						
		Emergency department visits	3.58 (5.8)	3.08 (3.7)	3.89 (6.8)	.45	
		Admissions	1.65 (2.2)	1.66 (2.2)	1.63 (2.2)	.88	
**Outcomes**							
	**Revisit type^b^, n (%)**						.37
		Emergency department visit	191 (39)	68 (37)	123 (40)		
		Readmission	302 (61)	117 (63)	185 (59)		
	**Predictability of revisit, n (%)**						.04
		Highly	115 (23)	47 (25)	68 (22)		
		Somewhat	142 (29)	58 (31)	84 (27)		
		Unknown	11 (2)	0 (0)	11 (4)		
		Not at all	228 (46)	80 (43)	148 (48)		
	**Revisit related to index admission, n (%)**						.09
		Directly	213 (43)	69 (37)	144 (46)		
		Indirectly	58 (12)	29 (16)	29 (9)		
		Unrelated	165 (33)	64 (35)	101 (32)		
		Unsure	60 (12)	23 (12)	37 (12)		
	Patient referred back to emergency department, n (%)		160 (32)	66 (36)	94 (30)	.21	
	Time from discharge to revisit, µ (SE)		12.77 (8.53)	15.23 (8.13)	11.31 (8.44)	<.001	

^a^n=462 (171 engaged in mHealth and 298 not engaged in mHealth).

^b^n=493 (185 engaged in mHealth and 308 not engaged in mHealth).

Patients who engaged with mHealth were younger (n=47, 26% aged <50 years vs n=41, 14% of those not engaged; *P*=.004 across age groups) and more likely to have commercial insurance (n=43, 23% vs n=35, 11%; *P*=.01). Patients in both groups had similar rates of ED visits and hospitalizations in the year prior to index admission (ED visits, mHealth users vs nonusers: 3.08 vs 3.89, *P*=.45; hospital admissions: 1.66 vs 1.63, *P*=.88).

### Outcomes

Across both groups, hospital readmissions were nearly twice as common as standalone ED returns (117/185, 63% of revisits among mHealth users vs 185/311, 59% among nonusers). Revisits were more predictable among patients who engaged with mHealth (105/185, 56% highly or somewhat predictable vs 152/311, 49% among non-mHealth users; *P*=.04). A similar proportion of revisits were directly or indirectly related to the index admission among mHealth users and nonusers (98/185, 53% vs 173/311, 55%; *P*=.09). mHealth users and nonusers had similar rates of ED referrals from their outpatient teams (66/185, 36% vs 94/311, 30%; *P*=.21). On average, those engaged with mHealth had more time between their index discharge and revisit (15.2 vs 11.3 days; *P*<.001).

### Intervisit Interactions

**Figure 1:**
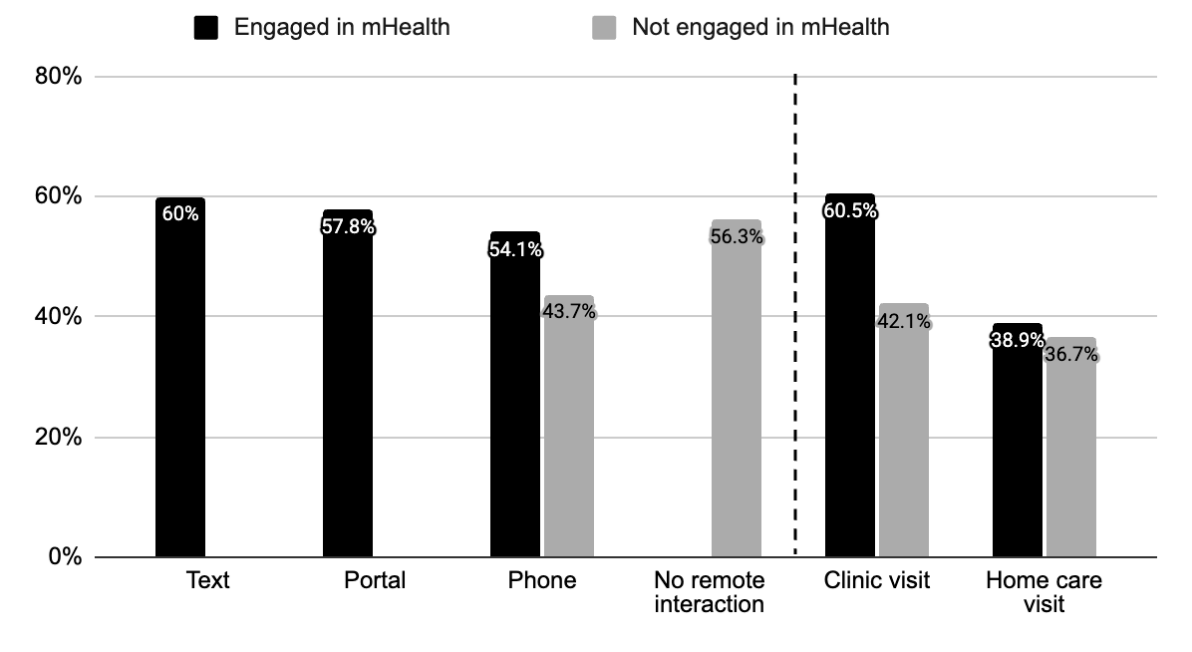
Summary of intervisit health care interactions. Categories are not mutually exclusive.*P value significant at *P*<.05 (only applied for interaction categories relevant to both study arms). mHealth: mobile health.

Among mHealth users, similar numbers of patients used SMS text messaging versus the patient portal (111/185, 60% vs 107/185, 58%; Figure 1, Multimedia Appendix 3). mHealth users were more likely than nonusers to engage with practices via non-mHealth modalities (telephone: 54% vs 44%, *P*=.03; clinic visits: 112/185, 61% vs 131/311, 42%; *P*<.001). Both groups had similar rates of home care visits (72/185, 39% vs 114/311, 37%; *P*=.60).

## Discussion

### Principal Findings

We examined patient, hospitalization, and intervisit health care interaction features among patients enrolled in an mHealth program, MORE-PC, with 30-day hospital revisits. We found that despite active outreach offered by the MORE-PC program, a minority of patients in the intervention arm used mHealth to communicate with outpatient providers before returning to the hospital. Notably, the parent MORE-PC trial found no significant reduction in 30-day readmissions, suggesting that the presence of an mHealth outreach program alone may be insufficient to reduce revisits without sufficient patient engagement. mHealth users tended to be younger and more likely to have commercial insurance. Predictability of revisits was high among those who used mHealth, and time from index discharge to revisit was longer as compared to those who did not use mHealth. In addition, those who used mHealth were also more likely to use other means of communication with outpatient providers.

Similar to our study, previous research has shown disparities in mHealth use by age, socioeconomic status, and insurer [17,18]. One potential explanation for the differences in usage by age may be that older patients have more difficulty using technology or be less inclined to adopt new technology [19,20]. Insurance and socioeconomic differences likely go hand in hand, and may be associated with mHealth use through mechanisms such as education or affordability of technology [18]. Interestingly, we did not find significant differences in mHealth use by race or ethnicity, though prior work has shown disparities by race and ethnicity [17,18]. With future development and expansion of mHealth interventions, it is crucial to consider ways of ensuring equitable implementation of mHealth interventions given early evidence of disparities across age, racial or ethnic, and socioeconomic groups.

Unsurprisingly, we found higher levels of revisit predictability among those who used mHealth. Though expected with greater communication with practices, this is encouraging as it suggests increased awareness of issues prompting a revisit. Though predictability may not have any bearing on preventability of revisits, timely identification of areas for potential intervention may help to avert a subset of revisits. In line with this, we found time from discharge to revisit was, on average, longer for mHealth users, suggesting mHealth use may delay but not prevent revisits. In line with previous work, we found no major difference in relatedness of index hospitalizations and revisits [21].

mHealth users interacted with outpatient teams by telephone and clinic visits at higher rates than nonusers. This raises concern that mHealth offerings may be additive, rather than substitutive, to traditional communication modalities, benefitting engaged groups more than reaching disengaged groups. For example, although it appears promising that mHealth use is associated with increased predictability and prolonged time to revisit, it is possible that this is more reflective of patient engagement than true advantages of mHealth. Previous research has explored such challenges, highlighting engagement of diverse patients for development of health applications and usage of community health workers to encourage uptake, though more work is needed in this area [22,23].

### Limitations

First, this study included patients within a single health system, and thus results may not be generalizable. Second, chart review methods may be subject to bias. We created data extraction templates and reviewed an initial sample as a group to try to minimize discrepancies across reviewers but recognize this bias cannot be entirely eliminated. Third, we categorized mHealth engagement as responding to SMS text messages or sending portal messages, but definitions of mHealth vary, and it is possible that those who engage via SMS text message may be different from those who engage via portal. Moreover, defining engagement through a binary variable may not capture meaningful differences across a spectrum of mHealth engagement. Finally, this study focuses on a single sample of mHealth users and nonusers with 30-day revisits but does not capture items such as patient satisfaction or strength of provider handoffs that may also be used to define success mHealth in transitions of care.

### Conclusions

In this secondary analysis of patients enrolled in an mHealth intervention who experienced 30-day revisits, mHealth engagement was limited. mHealth users were more likely to be younger, commercially insured, and to interact with their practices by traditional modalities (telephone and clinic visits), raising concerns about equity of mHealth interventions and their potential to increase disparities. We also found revisits among mHealth users occurred farther from their index discharge and were considered more predictable, suggesting potential benefits of mHealth engagement. Further research is needed to better define meaningful engagement with mHealth, identify outcomes most important and relevant to mHealth use, and understand how to leverage mHealth to equitably engage patients during transitions of care.

## Appendix

Multimedia Appendix 1 STROBE checklist.

Multimedia Appendix 2 Chart review extraction template.

Multimedia Appendix 3 Table of intervisit interactions.

## Abbreviations

ED: emergency department
mHealth: mobile health
MORE-PC: Mobile Outreach to Reduce Emergencies-Primary Care
STROBE: Strengthening the Reporting of Observational Studies in Epidemiology

## Data Availability

Data analyzed in this study are not publicly accessible due to inclusion of patient-level data from the electronic medical record.
