# Supplementary material for: Understanding mHealth Engagement Among Patients With 30-Day Hospital Revisits: Secondary Analysis of a Randomized Clinical Trial
Source: J Med Internet Res. 2026 May 29;28:e89067. doi: 10.2196/89067 (PMC13263652; doi:10.2196/89067)
Supplement: Multimedia Appendix 2 [file jmir_v28i1e89067_app2.docx]

**Appendix 2.** Chart Review Extraction Template

| MRN |  |
| --- | --- |
| Disch date |  |
| Revisit date |  |
| Discharge type (full adm, obs) |  |
| Revisit type (ED, obs, full adm) |  |
| Time from disch to revisit |  |
| Primary discharge diagnosis |  |
| Category of diagnosis |  |
| Primary revisit diagnosis |  |
|  |  |
| Was the revisit related to the index admission? (Directly, indirectly, maybe, unrelated) |  |
| Comment |  |
| Did the patient reach out via MORE-PC? (Yes, no) |  |
| Was the MORE PC escalation related to the revisit? (Yes, no, maybe) |  |
| Comment |  |
| Did the patient reach out through any other remote means? (Yes, no) |  |
| If yes, how? (Phone or portal) |  |
| Total types of communications (Text, Portal, Phone) |  |
| Were the communications related to the revisit? (Yes, no, somewhat) |  |
| Comment |  |
| Did the patient have an in person or telehealth visit prior to their revisit? (Yes, no) |  |
| Was anything mentioned in the visit related to their revisit? (Yes, no, somewhat) |  |
| Comment |  |
| Was this a planned readmission? (Yes, no) |  |
| Was the patient referred back to the hospital based on any of the notes? (Yes, no) |  |
| Based on encounter documentation, did the revisit seem predictable at any point prior to explicit ED referral or presentation to the ED? (Highly, somewhat, not at all) |  |
| If highly or somewhat, when did this become apparent? (At the time or discharge or between visits) |  |
| Please explain your reasoning for selecting level of predictability |  |
| Comment |  |
| Did the revisit seem preventable? (Yes, no, maybe) |  |
| If yes, how? |  |
| If no, why not? |  |
| Was anything done to try to avoid this readmission? (Yes, no) |  |
| If yes, what was done? |  |
| Comment |  |
| Does the patient or their family member engage with PennChart between visits? (Yes, no) |  |
| Patient died within 6 mos? |  |
| Does the pt have home care visits? (Yes, no) |  |
| Comments |  |
| Were the index and revisit locations the same? |  |
