# Supplementary material for: Understanding mHealth Engagement Among Patients With 30-Day Hospital Revisits: Secondary Analysis of a Randomized Clinical Trial
Source: J Med Internet Res. 2026 May 29;28:e89067. doi: 10.2196/89067 (PMC13263652; doi:10.2196/89067)
Supplement: Multimedia Appendix 3 [file jmir_v28i1e89067_app3.docx]

**Appendix 3.** Table of Inter-Visit Interactions*

| Interaction Type | Engaged in mHealth (n out of 185) | % | Not Engaged in mHealth (n = 311) | % |
| --- | --- | --- | --- | --- |
| Text | 111 | 60.0% | 0 | 0% |
| Portal | 107 | 57.8% | 0 | 0% |
| Phone | 100 | 54.1% | 136 | 43.7% |
| No Remote Interaction | 0 | 0% | 175 | 56.3% |
| Clinic Visit | 112 | 60.5% | 131 | 42.1% |
| Home Care Visit | 72 | 38.9% | 114 | 36.7% |

*Categories are not mutually exclusive
